# A Method for Combined Retinal Vascular and Tissue Oxygen Tension Imaging

**DOI:** 10.1038/s41598-017-10955-1

**Published:** 2017-09-06

**Authors:** Anthony E. Felder, Justin Wanek, Michael R. Tan, Norman P. Blair, Mahnaz Shahidi

**Affiliations:** 1 Department of Bioengineering, University of Illinois at Chicago, Chicago, IL 60607 USA; 2 Department of Ophthalmology and Visual Science, University of Illinois at Chicago, Chicago, IL 60612 USA; 3 Department of Ophthalmology, University of Southern California, Los Angeles, CA 90033 USA

## Abstract

The retina requires adequate oxygenation to maintain cellular metabolism and visual function. Inner retinal oxygen metabolism is directly related to retinal vascular oxygen tension (PO_2_) and inner retinal oxygen extraction fraction (OEF), whereas outer retinal oxygen consumption (QO_2_) relies on oxygen availability by the choroid and is contingent upon retinal tissue oxygen tension (tPO_2_) gradients across the retinal depth. Thus far, these oxygenation and metabolic parameters have been measured independently by different techniques in separate animals, precluding a comprehensive and correlative assessment of retinal oxygenation and metabolism dynamics. The purpose of the current study is to report an innovative optical system for dual oxyphor phosphorescence lifetime imaging to near-simultaneously measure retinal vascular PO_2_ and tPO_2_ in rats. The use of a new oxyphor with different spectral characteristics allowed differentiation of phosphorescence signals from the retinal vasculature and tissue. Concurrent measurements of retinal arterial and venous PO_**2**_, tPO_2_ through the retinal depth, inner retinal OEF, and outer retinal QO_**2**_ were demonstrated, permitting a correlative assessment of retinal oxygenation and metabolism. Future application of this method can be used to investigate the relations among retinal oxygen content, extraction and metabolism under pathologic conditions and thus advance knowledge of retinal hypoxia pathophysiology.

## Introduction

The retinal tissue requires an adequate supply of oxygen for cellular metabolism and function. Retinal ischemia due to reduced blood flow has been implicated in the development of vision threatening pathologies such as neovascularization and macular edema^1, 2^. Furthermore, inadequate oxygen availability can lead to hypoxic injury and eventual cell death. Therefore, assessment of retinal oxygenation is essential to improve knowledge of disease pathophysiology and advance treatments that target alleviation of hypoxia-induced pathologies.

Several techniques have become available for quantitative assessment of oxygen content within the retinal vasculature or tissue. Specifically, retinal oximetry for measurement of hemoglobin oxygen saturation (SO_2_) has been performed by spectrophotometry^3–5^, photoacoustic ophthalmoscopy^6^, and visible optical coherence tomography^7, 8^. Additionally, retinal vascular oxygen tension (PO_2_) has been reported using phosphorescence lifetime imaging^9–11^. Direct depth-resolved measurements of retinal tissue oxygen tension (tPO_2_) have been provided by the oxygen-sensitive microelectrode technique at single point locations^12–14^ and by phosphorescence lifetime imaging at multiple contiguous locations^15^. Furthermore, information about the metabolic activity of the retinal tissue has become available by calculation of inner retinal oxygen extraction fraction (OEF) based on retinal vascular oxygen content^5, 16^ and by estimation of outer retinal oxygen consumption (QO_**2**_) from retinal tPO_2_ depth profiles^14, 17, 18^.

To date, these parameters of retinal oxygenation and metabolism have been measured independently in separate animals. Thus, current techniques precluded the assessment of relations among these parameters in the same animal under physiological or pathological conditions which is essential to improve understanding of retinal ischemia pathophysiology. Retinal hypoxia is implicated in the development of vision-threatening retinal pathologies, yet there are currently no direct methods to measure tPO_2_ in humans. Alterations in inner retinal OEF due to hypoxia^16^ and retinal disease^19^ have been demonstrated, but the relationship between tPO_2_ and OEF is not known. Furthermore, although presumed, a correspondence between inner retinal tPO_2_ and venous PO_2_ has not been established. Measurements of these parameters in separate animals cannot accurately determine the association between them due to physiological variations among animals. Therefore, concurrent measurements of retinal vascular PO_2_ and tPO_2_ in the same animals are necessary to infer reduced tPO_2_ based on altered OEF or venous PO_2_ under retinal ischemic conditions. These findings may be translated to humans to assess retinal hypoxia and identify OEF thresholds necessary to sustain tPO_2_. The purpose of the current study is to report an innovative optical system for dual oxyphor phosphorescence lifetime imaging to near-simultaneously measure retinal vascular PO_2_ and tPO_2_ in rats, derive OEF and QO_**2**_, and determine associations among these parameters.

## Methods

### Animals

All experimental procedures were in compliance with the ARVO Statement for the Use of Animals in Ophthalmic and Vision Research and approved by the Animal Care Committee of the University of Illinois at Chicago. The study was performed in 10 Long Evans pigmented rats. Rats were anesthetized with intraperitoneal injections of ketamine (100 mg/kg) and xylazine (5 mg/kg) and additional injections were given as required to maintain anesthesia. One day prior to imaging, oxyphor G2 (Oxygen Enterprises, Philadelphia, PA) was constituted at 1.5 μM and administered as a 5 μL intravitreal bolus injection for retinal tPO_2_ imaging. Immediately prior to imaging, oxyphor R0 (Frontier Scientific, Logan, Utah) was administered intravenously (20 mg/kg) for retinal vascular PO_2_ imaging. Before imaging, pupils were dilated with 2.5% phenylephrine and 1% tropicamide, and a glass cover slip with 1% hydroxypropyl methylcellulose was placed on the cornea to minimize corneal refractive power and prevent dehydration. Rats were placed on an animal holder with a closed-loop water heater to maintain body temperature at 37 °C and were spontaneously breathing during imaging. One eye of each rat was imaged in temporal or nasal regions within three-disk diameters (600 microns) from the edge of the optic nerve head.

### Phosphorescence Lifetime Imaging

**Figure 1:**
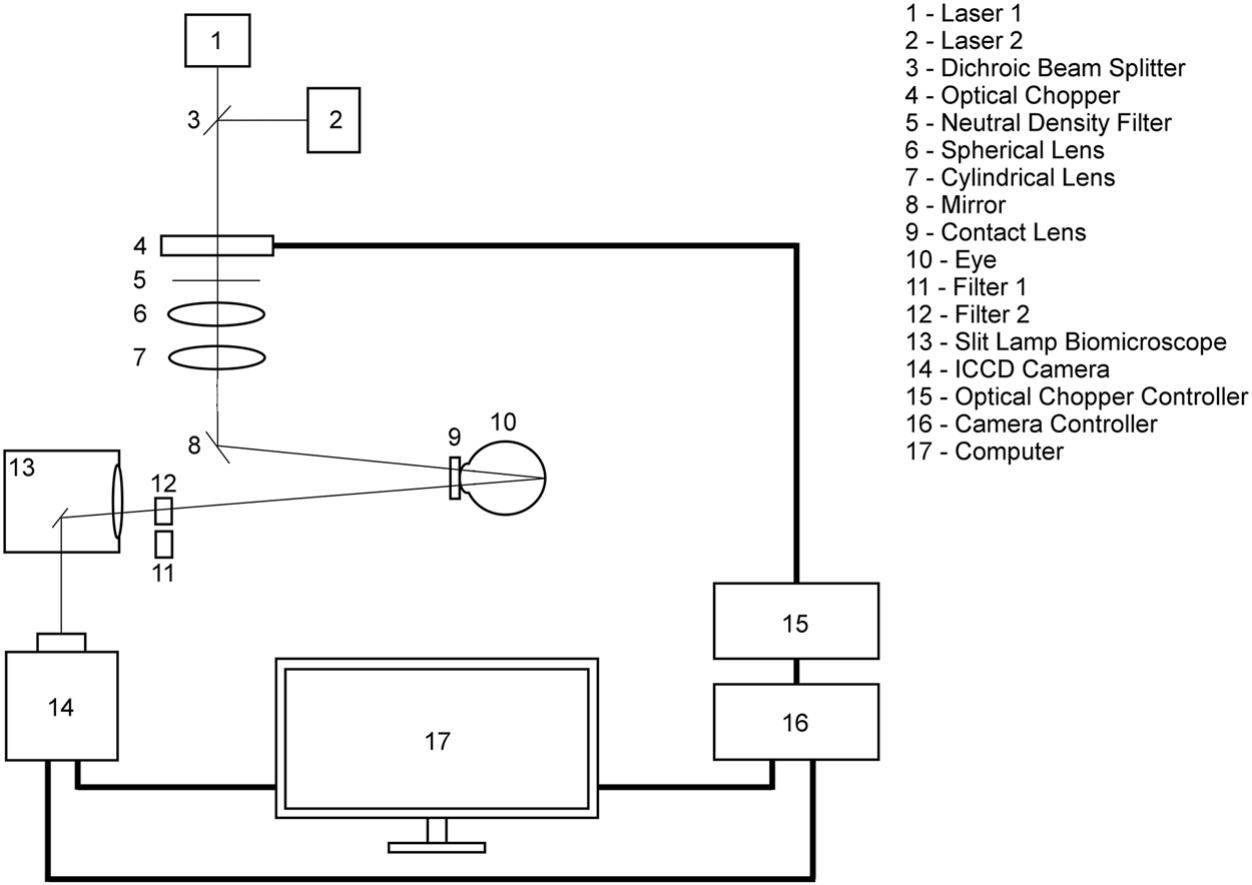
Schematic diagram of the optical imaging system for near-simultaneous measurement of retinal vascular and tissue oxygen tension. Thick lines represent physical connections between system components and thin lines represent the optical path.

Our previously established optical imaging system^9, 15^ for either retinal vascular PO_2_ or tPO_2_ measurement was modified for near-simultaneous measurement of both parameters by dual oxyphor phosphorescence lifetime imaging (Fig. 1). The use of two oxyphors (R0 and G2) with different absorption and emission spectra allowed differentiation of phosphorescence signal from within the retinal vasculature and tissue. Two diode lasers at 532 nm (Lasermate Group, Inc. MGM-10) and 635 nm (Lasermate Group, Inc. LTC6358AH) were incorporated into the imaging system for excitation of R0 and G2 oxyphors, respectively. The power of each laser was adjusted to 120 µW at the cornea. Both lasers were projected at an oblique angle to the imaging axis and focused to a co-localized 1 mm vertical line on the retina. Since the incident lasers were not coaxial with the imaging path, phosphorescence emission through the retinal depth appeared laterally displaced on the optical section phosphorescence image. A high pass (>650 nm; Thorlabs, Inc.) or bandpass filter (810 ± 25 nm; Midwest Optical Systems, Inc.) was placed interchangeably in the imaging path to selectively image the phosphorescence emission of R0 (within the retinal vasculature) or G2 (within the retinal tissue), respectively. Both lasers were modulated by an optical chopper at 1.6 kHz. Optical section phosphorescence images were acquired by an intensified charge-coupled device (ICCD) camera, while the gain of the intensifier was modulated by the camera software at the same frequency. The optical chopper frequency, ICCD gain modulation frequency and temporal delay increments between the two were selected to produce phase shifts between 0° and 180°. Phosphorescence lifetime was measured from 10 phase-delayed optical section phosphorescence images, as previously described^9, 15^.

Image acquisition consisted of imaging G2 and R0 phosphorescence sequentially. First, three repeated phase-delayed optical section phosphorescence images from the retinal tissue were acquired. Then, the excitation laser and emission filter were changed manually (within two seconds) and three repeated phase-delayed optical section phosphorescence images from the retinal vasculature were acquired at exactly the same retinal location. The total time for image acquisition was less than 60 seconds.

### Vascular PO_2_ and Inner Retinal OEF

Vascular PO_2_ in major retinal arteries and veins was measured from the phosphorescence lifetime using the Stern-Volmer equation and the R0 oxyphor’s quenching constant and lifetime in a zero-oxygen environment, as previously described^9, 20^. PO_2_ in each blood vessel was calculated by averaging three repeated measurements. A mean arterial and venous PO_2_ (PO_2A_ and PO_2V_) was determined for each rat by averaging measurements in each vessel type. Inner retinal OEF was calculated from PO_2A_ and PO_2V_ and using the oxyhemoglobin dissociation curve in rat^21^, as previously described^16^. Inner retinal OEF is the fraction of oxygen supplied by the retinal circulation that is extracted by the inner retinal tissue for metabolism, or equivalently, the ratio of inner retinal oxygen metabolism to inner retinal oxygen delivery.

### Retinal tPO_2_ and Outer Retinal QO_2_

Retinal tissue optical section phosphorescence images were first processed to minimize retinal curvature, then smoothed in vertical (y-axis) and axial (z-axis) dimensions using a 2D anisotropic averaging filter (6 × 4 pixels), corresponding to 18 μm in both axes. A depth-resolved tPO_2_ image was generated from the phosphorescence lifetime measured at each pixel using the Stern-Volmer equation and the G2 oxyphor’s quenching constant and lifetime in a zero-oxygen environment^22^, as previously described^15^. A mean depth-resolved retinal tPO_2_ image was generated from three repeated images.

Along the vertical dimension of each retinal tPO_2_ image (superior to inferior), 35 contiguous tPO_2_ depth profiles were generated by vertically averaging tPO_2_ over 10 pixels (30 microns) and plotting tPO_2_ as a function of retinal depth, as previously described^15^. The outer and inner retina were defined as 50% to 100% and 0% to 50% of the retinal depth, respectively. Maximum outer retinal tPO_2_, minimum outer retinal tPO_2,_ and mean inner retinal tPO_2_ were calculated from each tPO_2_ depth profile. Mean inner retinal tPO_2_ was plotted along the vertical dimension of the image to demonstrate changes in this parameter with respect to retinal arteries and veins. Furthermore, mean values for each parameter were calculated from all profiles along the tPO_2_ image.

From each retinal tPO_2_ depth profile, outer retinal QO_2_ was calculated by fitting a three-layer, one-dimensional, steady-state oxygen diffusion model using a non-linear least squares technique, as previously described^12, 14, 17, 23^. In this model, oxygen diffuses in one dimension from the choroidal circulation across the outer retinal depth, which is divided into three layers^14^, based on their oxygen consumption properties. QO_2_ has been shown to be negligible in layers 1 and 3^23^, which correspond to the photoreceptor outer segments and outer nuclear layer, respectively. In contrast, oxygen is consumed in layer 2 by mitochondria of the photoreceptor inner segments, yielding a quadratic relationship between tPO_2_ and retinal depth. A mean outer retinal QO_2_ was calculated by averaging measurements obtained from all tPO_2_ depth profiles along the vertical dimension of the tPO_2_ image.

### Data availability

The data generated are available from the corresponding author on reasonable request.

## Results

**Figure 2:**
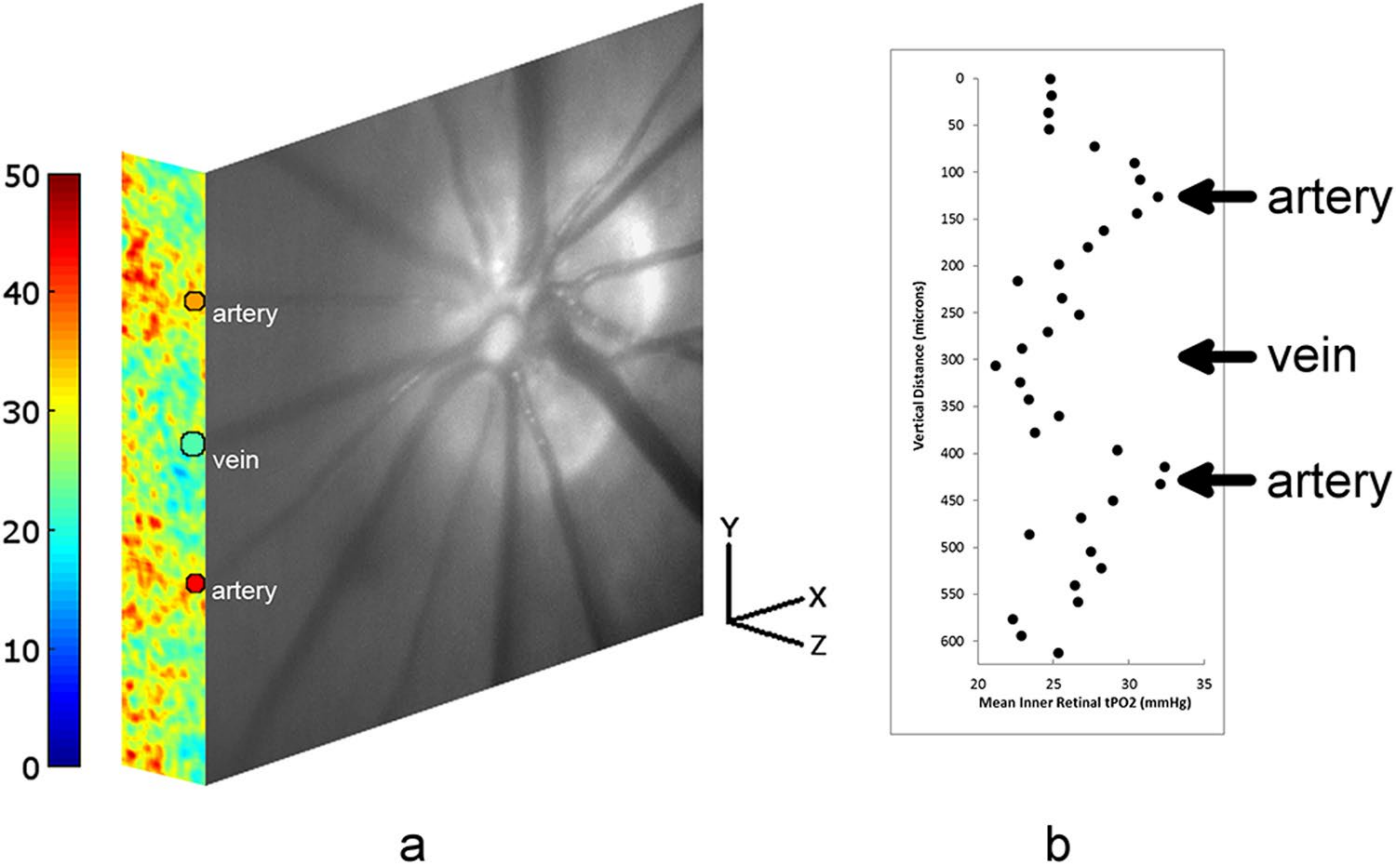
(**a**) Combined depth-resolved retinal vascular oxygen tension (PO_2_) and tissue oxygen tension (tPO_2_) images overlaid on the retinal reflectance image in a rat. The vascular PO_2_ image displays measurements in two arteries and one vein (labeled), outlined by black circles. Color bar indicates PO_2_ in mmHg. (**b**) Inner retinal tPO_2_ plotted along the vertical dimension (superior to inferior) along the tPO_2_ image. Locations of the two arteries and one vein are marked.

A representative example of a retinal vascular PO_2_ and tPO_2_ image obtained at the same location overlaid on the retinal reflectance image is shown in Fig. 2a. The vascular PO_2_ image displays measurements in two arteries and one vein, demonstrating higher PO_2A_ than PO_2V_, as expected. The tPO_2_ image shows higher tPO_2_ near the chorioretinal interface compared to inner retina. Mean inner retinal tPO_2_ plotted along the vertical dimension (superior to inferior) is shown in Fig. 2b, displaying higher tPO_2_ near arteries than the vein.

**Figure 3:**
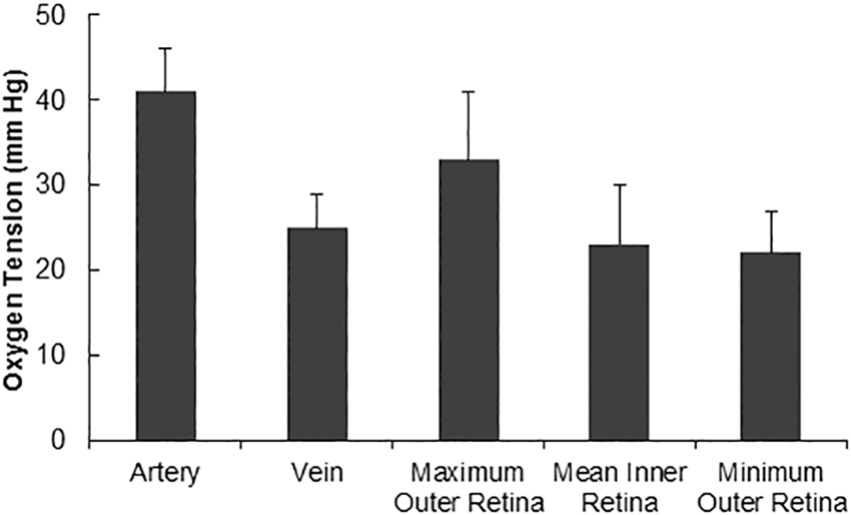
Retinal arterial and venous oxygen tension (PO_2_) and tissue oxygen tension (tPO_2_) measurements in 10 rats. Error bars indicate standard deviations.

**Figure 4:**
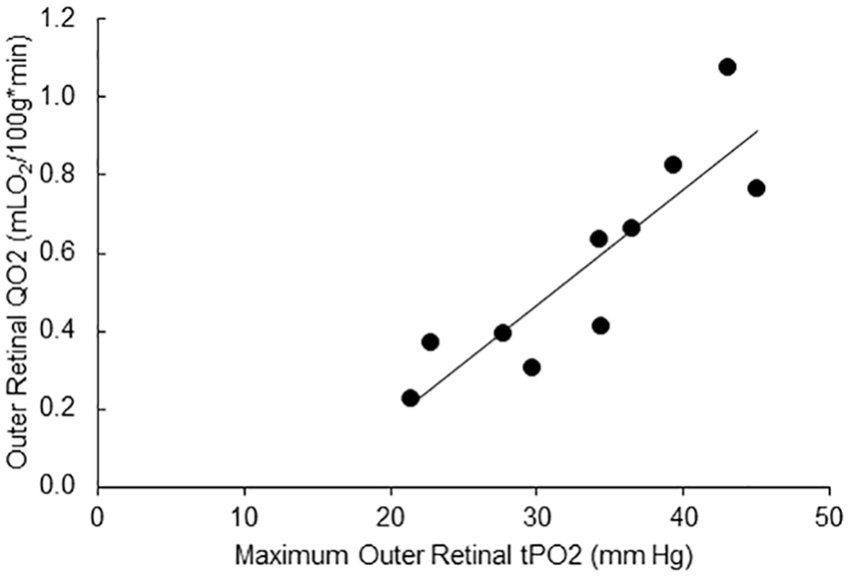
Relationship between outer retinal oxygen consumption (QO_2_) and maximum outer retinal tissue oxygen tension (tPO_2_). The regression line best fit to the data is shown (R^2^ = 0.77).

Compiled retinal vascular PO_2_ and tPO_2_ measurements in all rats are presented in Fig. 3. PO_2A_ and PO_2V_ were 41 ± 5 mmHg and 25 ± 4 mmHg, respectively (P < 0.001; N = 10). Inner retinal tPO_2_, minimum outer retinal PO_2_, maximum outer retinal PO_2_ were 23 ± 7 mm Hg, 22 ± 5 mm Hg and 33 ± 8 mm Hg, respectively. There was no significant difference between PO_2V_ and mean inner retinal tPO_2_ (P = 0.4). Inner retinal OEF was 0.58 ± 0.11 and outer retinal QO_2_ was 0.57 ± 0.27 mLO_2_/100 g*min. There was no significant correlation between inner retinal OEF and mean inner retinal tPO_2_ (R^2^ = 0.05; P = 0.53; N = 10). As demonstrated in Fig. 4, there was a significant correlation between outer retinal QO_2_ and maximum outer retina tPO_2_ (R^2^ = 0.77; P < 0.001; N = 10).

## Discussion

A novel optical imaging method for near-simultaneous imaging of retinal vascular PO_2_ and tPO_2_ was demonstrated by phosphorescence lifetime imaging of dual oxyphors delivered intravenously and intravitreally. For the first time, concurrent assessment of oxygen content across the retinal depth and oxygen metabolism metrics of the inner and outer retina was demonstrated. Future application of this method can provide knowledge of relations among these parameters under experimental pathologic conditions and thus yield a comprehensive understanding of retinal oxygenation and metabolism dynamics.

Measurements of retinal PO_2A_ and PO_2V_ were in general agreement with those from previous studies^24, 25^. Depth-resolved retinal tPO_2_ images displayed maximum tPO_2_ near the choroid, consistent with normal physiology. Inner retinal tPO_2_ measurements using the G2 oxyphor were similar to our previously published data with the R2 oxyphor^15^ and those reported by the oxygen microelectrode technique^14^. Alterations in inner retinal tPO_2_ were consistent with the nearby presence of arteries and veins. Inner retinal OEF was 0.58, ﻿on average, indicating 58% of the oxygen delivered by the retinal circulation was extracted for metabolism by the inner retinal tissue. The mean inner retinal OEF was higher as compared to our previous study^16^ and maximum outer retinal tPO_2_ in the current study was slightly lower than previous reports^14, 15^. Both of these results are consistent with the presence of reduced systemic oxygenation^26^, which likely resulted from the effect of anesthesia while the rats were under spontaneous breathing conditions^27^. Furthermore, the variability of tPO_2_ adjacent to the chorioretinal interface along the tPO_2_ image may, at least in part, be attributed to presumed anesthesia-induced systemic hypotension. This may have resulted in reduced choroidal blood flow, thus altering tPO_2_ gradients within the retinal depth. Overall, the results demonstrate the ability of the optical imaging system to measure retinal vascular PO_2_, tPO_2_ and inner retinal OEF.

Measurements of outer retinal QO_2_ obtained in the current study were similar to our previously reported values^17^, but lower than those obtained by the oxygen microelectrode technique in light-adapted rats^12, 14, 18^. This difference is likely due to intraretinal phosphorescence scattering which decreases the depth resolution, minimizes the curvature of tPO_2_ profiles and calculated values of QO_2_. Nevertheless, outer retinal QO_2_ was significantly associated with maximum outer retinal tPO_2_, in agreement with a previous study^14^, though a small portion of oxygen utilized by the photoreceptors is supplied by the retinal vasculature^14^. This result suggests a dependence of the photoreceptor metabolic function on the level of oxygen supplied by the choroidal circulation.

Simultaneous measurements of retinal vascular PO_2_ and tPO_2_ coupled with derivation of inner retina OEF and outer retinal QO_2_ can advance knowledge of retinal oxygen dynamics. For example, we demonstrated here, for the first time, that there was no significant correlation between inner retinal OEF and mean inner retinal tPO_2_ under healthy condition. This result suggests that despite physiological variations, tPO_2_ is well-maintained due to compensatory alterations in OEF and oxygen delivery, and implies the presence of highly effective regulatory mechanisms. Previous studies have shown alterations in OEF under experimental hypoxia in rats^16^. In future studies, by relating inner retinal tPO_2_ with OEF under graded hypoxia/ischemia, diabetes or light flicker stimulation, the OEF threshold necessary to sustain tPO_2_ may be identified. Additionally, data obtained under graded levels of ischemia may be used to establish a relationship between inner retinal tPO_2_ and PO_2V_. Furthermore, combined retinal blood flow measurements with the current method can be used to determine the inner retinal oxygen delivery threshold necessary to maintain retinal tPO_2_ with direct relevance to retinal ischemic diseases. These thresholds can only be accurately determined with concurrent retinal vascular PO_2_ and tPO_2_ measurements in the same animal.

One potential limitation of this method is the scattering of phosphorescence light within the retina and vitreous which can degrade image quality. However, compared to the R2 oxyphor, less scattering is expected from the G2 oxyphor due to its longer phosphorescence emission wavelength. The quenching constants used to calculate PO_2_ were obtained from literature and may be different within the retinal tissue environment. Phototoxicity may affect data derived from images acquired repeatedly at the same location with R0 oxyphor^28^, but any effect of phototoxicity on measurements derived using the G2 oxyphor is not known. Although, the laser irradiance was below the threshold for tissue damage, repeatability of measurements obtained at the same location with the use of dual oxyphors will need further evaluation. The feasibility of the method was demonstrated in a limited number of rats and future studies using larger sample sizes are needed to fully establish the utility of the method.

In conclusion, for the first time, near-simultaneous measurement of retinal vascular and tissue oxygen tension by phosphorescence lifetime imaging was demonstrated. Future application of this method under challenged physiologic or pathologic conditions permits correlation of retinal vascular and tissue oxygen content and can potentially elucidate retinal oxygenation dynamics.
